# Analysis of ultrasound and magnetic resonance imaging characteristics of kaposiform hemangioen dothelioma

**DOI:** 10.1186/s40644-024-00801-9

**Published:** 2024-11-07

**Authors:** Chuang Li, Zhimeng Shen, Qi Sun, Gang Wu

**Affiliations:** 1 Department of Ultrasound, Henan Provincial People’s Hospital, No. 7 Weiwu Road, Zhengzhou City, 450000 Henan Province China; 2 Department of Radiology, The First Affiliated Hospital of Zhengzhou University, Zhengzhou, China; 3 Department of Pathology, Henan Provincial People’s Hospital, Zhengzhou, China

**Keywords:** Kaposiform hemangioen dothelioma, Kasabach-Merritt phenomenon, Ultrasound, Magnetic resonance imaging, Clinical characteristics

## Abstract

**Objective:**

The present study aims to investigate the ultrasound and magnetic resonance imaging (MRI) characteristics of kaposiform hemangioen dothelioma (KHE).

**Methods:**

A retrospective analysis was conducted on the clinical data of children diagnosed with KHE through postoperative pathology. Patients were divided into two groups: the KHE group and the KHE with Kasabach-Merritt Phenomenon (KMP) group (KMP group). Laboratory indicators, ultrasound, and MRI data were collected and analyzed statistically to summarize the imaging characteristics of the disease.

**Results:**

The levels of platelets and fibrinogen in the KHE group were significantly higher than those in the KMP group, while D-dimer levels, prothrombin time, and activated partial thromboplastin time were lower (*P* < 0.05). Ultrasound characteristics comparison revealed that lesions extending to the fat layer (42.47% vs. 54.24%) and invading the muscle layer (38.36% vs. 69.49%) were less common in the KHE group compared to the KMP group, with the lesion diameter being smaller in the KHE group (*P* < 0.05). The Adler grading predominantly showed Grade II (45.21%) in the KHE group, whereas Grade III (93.22%) was more prevalent in the KMP group (*P* < 0.05). MRI analysis indicated that the incidence of lesions invading the muscle layer and the presence of flow voids were lower in the KHE group compared to the KMP group (*P* < 0.05).

**Conclusion:**

KHE patients with KMP exhibit lesions that are more prone to extending into the fat layer and invading the muscle layer, with larger diameters and abundant blood flow. Additionally, the MRI images of the lesions may exhibit flow voids.

## Introduction

Kaposiform hemangioen dothelioma (KHE) is a rare vascular tumor [1] with local invasive growth, classified as a special type of hemangioma [2]. It predominantly occurs in infants and young children, affecting the skin and multiple organs [3]. Large lesions or those located deep within tissues can lead to life-threatening thrombocytopenia, known as Kasabach-Merritt phenomenon (KMP) [4]. The natural mortality rate of KHE ranges from 12–30% [5], and this rate increases to 20–30% when KHE is complicated by KMP, posing a significant threat to the lives of infants and young children. The residual complications also severely impact the children’ physical and mental health [6].

KHE exhibits a histopathological architecture characterized by richly dense, coalesced clusters of vascular cells, predominantly consisting of spindle cells encircling round epithelioid cells, which collectively manifest a benign morphology often resembling glomeruloid structures [7]. KHE has the potential to histologically mimic other soft tissue vascular tumors of different behaviors [8], such as tufted angioma, infantile hemangioma, and Kaposi’s sarcoma, necessitating vigilant differential diagnosis in clinical practice. Currently, clinical diagnostic methods for KHE mainly include ultrasound examination, CT, MRI, and pathological examination [9]. Ultrasound proves instrumental in diagnosing pediatric soft tissue vascular abnormalities, assessing lesion extent, evaluating complications, and monitoring treatment response [10]. MRI stands as a pivotal diagnostic modality for KHE, particularly when lesions present with ill-defined boundaries or infiltrative growth vascular masses associated with Kasabach-Merritt phenomenon [11]. Early diagnosis of KHE is crucial for improving the survival prognosis of affected children.

Given the primary focus of current research on KHE’s ultrasound, CT, and pathological characteristics, MRI remains a critical examination method. Therefore, this study undertakes a retrospective analysis of data from 132 KHE cases to summarize the ultrasound characteristics and MRI manifestations of KHE, thereby furnishing a solid foundation for precise clinical diagnosis.

## Materials and methods

### Clinical data

**Fig. 1:**
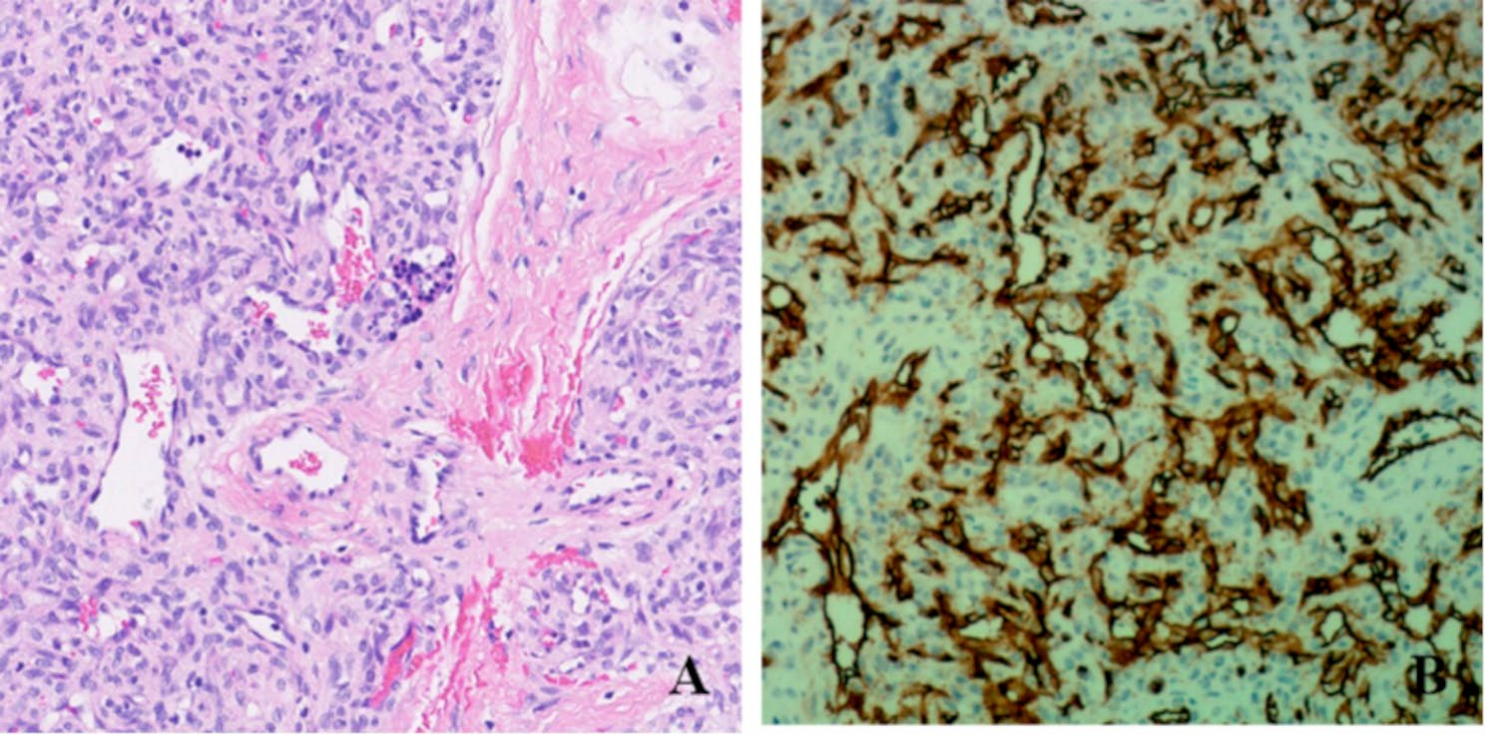
Histological presentation of KHE (**A**, ×200) and immunohistochemical results (**B**, ×200)

**Fig. 2:**
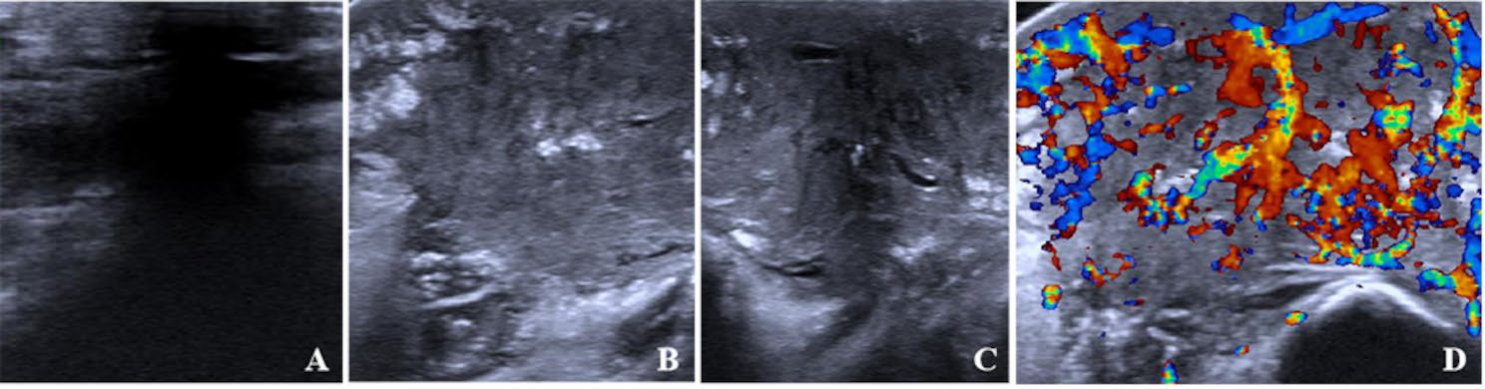
Ultrasound images. **A**: Ultrasound examination reveals predominantly hypoechoic internal echoes within the lesion; **B**: The lesion is predominantly hyperechoic internally; **C**: Tubular anechoic areas are visible within the lesion; **D**: Rich blood flow signals with a dendritic distribution are observed within the lesion (Adler grade III)

A retrospective analysis was performed on the clinical data of 132 children diagnosed with KHE through postoperative pathology and treated in our hospital from January 2014 to July 2024. Patients were divided into two groups: the KHE group and the KHE with KMP group (KMP group) based on the presence of KMP (platelet count < 50 × 10⁹/L). The inclusion criteria were as follows: (1) Infants and young children with subcutaneous hemangioma or congenital subcutaneous hemangioma diagnosed according to the “Diagnosis and Treatment Guideline for Hemangiomas and Vascular Malformations (2024 version),” [12] with KHE confirmed through postoperative pathology (Fig. 1); (2) patients underwent ultrasound and MRI examinations with clear images; and (3) availability of comprehensive clinical data. The exclusion criteria were as follows: (1) lesions exceeding 5.6 cm in diameter, precluding full visualization within a single image frame; (2) lesions located too superficially and easily diagnosable based on appearance; and (3) history of surgical, laser, or sclerotherapy interventions. This study was approved by the ethics committee of our hospital, with informed consent waived. This paper was approved by the ethics committee of our hospital, and all patients or their families signed informed consent forms before examination.

### Study method

#### General data

Clinical data including the patient’s gender, age at onset, and the location of the lesion (head and neck, trunk, limbs) were collected.

#### Laboratory tests

Data were collected on routine blood tests, platelet count (×10^9^/L), fibrinogen (g/L), D-dimer (µg/L), prothrombin time (s), and activated partial thromboplastin time (s).

#### Ultrasound examination

The ACUSON Sequoia ultrasound diagnostic system with an 18 L6 linear array probe, operating at a frequency range of 6–18 MHz, was utilized. The examination area was fully exposed, with the probe gently placed on the skin surface over the lesion. A thick layer of coupling gel was applied to the protruding lesion to create an acoustic window, centering the lesion in the field of view and adjusting the gain accordingly. Two-dimensional and color Doppler ultrasound examinations were conducted in a calm state of the child, observing and documenting internal echoes within the lesion (hypoechoic; mixed echoes; hyperechoic), echoes from surrounding adipose tissue (unchanged; enhanced; reduced), extension into the adipose layer, lesion diameter, muscle layer invasion, clarity of lesion boundaries, presence of anechoic tubular structures within the mass, and Adler blood flow grading (Grade 0; I; II; III) (Fig. 2). Two sonographers with more than ten years of experience interpreted the examination results.

#### MRI examination

**Fig. 3:**
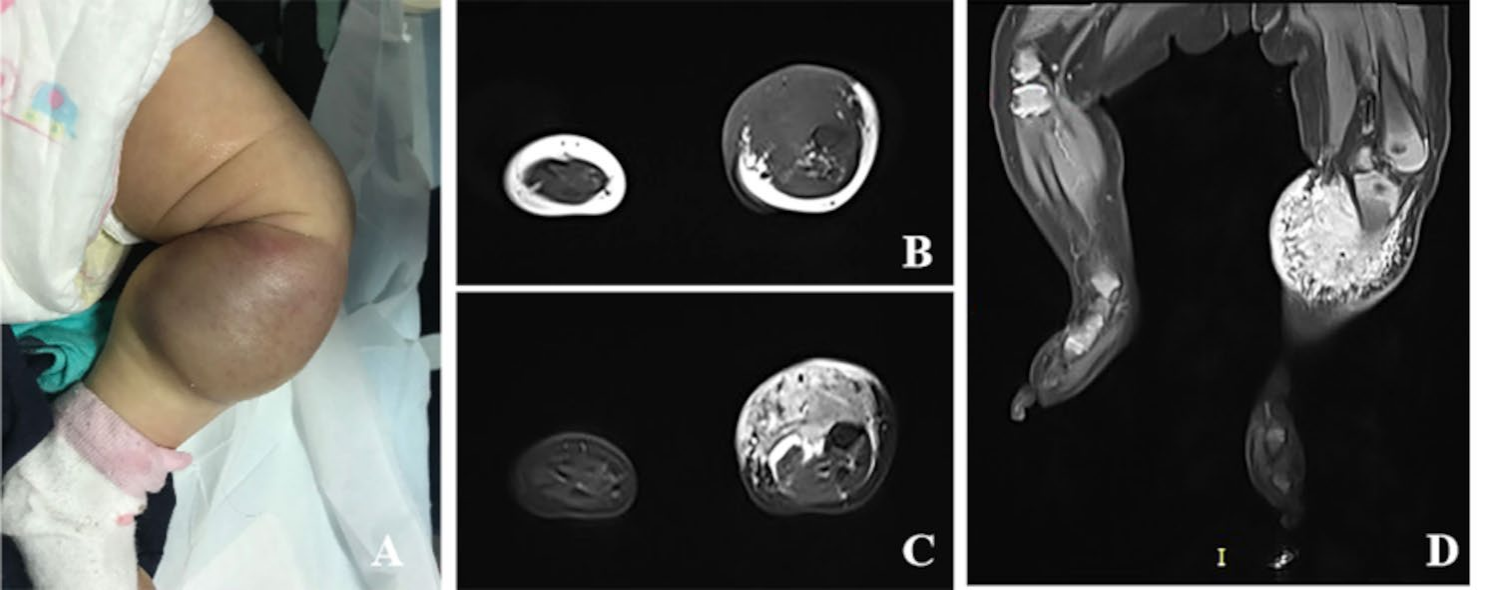
MRI images. **A**: Appearance of KHE in a 2-month-old female infant’s left leg; **B**: Coronal T1WI of the left leg shows a low-signal soft tissue mass surrounding the middle segment of the humerus; **C**: PDWI shows the mass to be hyperintense; **D**: Coronal contrast-enhanced FS T1WI reveals marked heterogeneous enhancement of the mass

A Siemens Magnetom Essenza 1.5T MRI scanner with an abdominal phased-array or body coil was used for the examination. The child was positioned supine, and after a plain scan, gadopentetate dimeglumine contrast agent (0.10 mmol/kg body weight) was administered intravenously at a rate of 2 mL/s through the cubital vein. Axial contrast-enhanced images were acquired, with additional coronal or sagittal fat-suppressed (FS) turbo spin-echo (TSE) T1-weighted scans depending on the lesion’s location. Parameters were as follows: T1WI: TR 630 ms, TE 20 ms; T2WI: TR 2600 ms, TE 62 ms; proton density-weighted imaging (PDWI): TR 2200 ms, TE 30 ms; diffusion-weighted imaging (DWI): TR 6000 ms, TE 976 ms, b-values = 0, 600 s/mm²; with a slice thickness of 5 mm, inter-slice gap of 1 mm, and FOV of 200 mm × 220 mm. Two senior radiologists independently reviewed the images in a blind manner, assessing the clarity of lesion boundaries, muscle layer invasion, presence of flow voids, T1WI signal (low; iso; high), and T2WI signal (low; iso; high) (Fig. 3).

### Statistical analysis

All data were statistically analyzed using the SPSS 26.0 software (SPSS Inc., Chicago, IL, USA). Normally distributed measurement data were expressed as mean ± standard deviation (x ± s), and comparisons between groups were made using the independent sample t-test. Non-normally distributed measurement data were expressed as median (P_25_, P_75_), and comparisons between groups were made using the Mann-Whitney U test. Enumeration data were expressed as counts (percentages) [n(%)], and comparisons between groups were made using the χ² test. A *P*-value < 0.05 was considered a statistically significant difference.

## Results

### Clinical data

A total of 132 pediatric patients with KHE were included in the study, comprising 65 males and 67 females, with ages at onset ranging from 1 to 144 months. Lesions were located in the head and neck in 30 cases, the trunk in 40 cases, and the limbs in 62 cases. The platelet count was 287.42 ± 124.83 × 10^9^/L, fibrinogen was 1.82 ± 0.70 g/L, D-dimer was 0.73 (0.24, 1.47) µg/L, prothrombin time was 11.94 ± 0.74 S, and activated partial thromboplastin time was 33.69 ± 13.29 S.

### Comparison of clinical data between KHE and KMP groups

Patients were divided into the KHE group (73 cases) and the KMP group (59 cases) based on the presence of KHE. Laboratory indices comparison revealed that the KHE group had higher platelet levels (289.66 ± 102.26 vs. 73.80 ± 28.18) and fibrinogen levels (2.08 ± 0.64 vs. 1.53 ± 0.81) than the KMP group, with statistically significant differences (*P* < 0.05). The KHE group had lower D-dimer levels [0.59 (0.40, 1.21) vs. 4.90 (1.89, 9.34)], prothrombin time (12.66 ± 1.43 vs. 15.69 ± 4.17), and activated partial thromboplastin time (36.94 ± 8.05 vs. 43.83 ± 9.92) compared to the KMP group, with statistically significant differences (*P* < 0.05) (Table 1).

**Table 1:** Comparison of laboratory indicators between the two groups of children

Indicators	KHE group (*n* = 73)	KMP group (*n* = 59)	χ^2^/t/Z	*P* value
Gender (Male)	36(49.32)	29(49.15)	0.941	0.832
Age (Months)	4(2, 6.50)	3(1, 6)	0.536	0.426
Platelets (×10^9^/L)	289.66 ± 102.26	73.80 ± 28.18	23.904	0.001
Fibrinogen (g/L)	2.08 ± 0.64	1.53 ± 0.81	0.794	0.021
D-dimer (µg/L)	0.59(0.40, 1.21)	4.90(1.89, 9.34)	0.874	0.013
Prothrombin time (S)	12.66 ± 1.43	15.69 ± 4.17	2.924	0.037
Activated partial thromboplastin time (S)	36.94 ± 8.05	43.83 ± 9.92	7.326	0.042

### Comparison of ultrasound characteristics between KHE and KMP groups

The proportion of lesions extending into the adipose layer (42.47% vs. 54.24%) and invading the muscle layer (38.36% vs. 69.49%) was lower in the KHE group compared to the KMP group, and the lesion diameter was smaller in the KHE group, with statistically significant differences (*P* < 0.05). The main blood flow grading in the KHE group was Grade II (45.21%), followed by Grade III (45.10%), while in the KMP group, it was primarily Grade III (93.22%), with statistically significant differences between the two groups (*P* < 0.05) (Table 2).

**Table 2:** Comparison of ultrasound characteristics between the two groups of children

Characteristic	KHE group (*n* = 73)	KMP group (*n* = 59)	χ^2^/Z	*P* value
Internal echo of the lesion			7.903	0.074
Hypoechoic	42(57.53)	28(47.46)		
Mixed echo	20(27.40)	14(23.73)		
Hyperechoic	11(15.07)	7(11.86)		
Fatty tissue echo			3.682	
No change	25(34.25)	14(23.73)		0.053
Enhanced	46(63.01)	45(76.27)		
Decreased	2(2.74)	0		
Extension towards the fat layer	31(42.47)	32(54.24)	2.902	0.042*
Lesion diameter	44.00(29.00,57.20)	63.00(50.00, 83.00)	5.313	0.012*
Invasion of the muscle layer	28(38.36)	41(69.49)	6.201	0.001*
Lesion boundary			1.932	0.132
Clear	12(16.44)	9(15.25)		
Blurry	61(83.56)	50(84.75)		
Tubular anechoic mass	43(58.80)	40(67.80)		
Adler grade			3.482	0.008*
I	10(13.70)	0		
II	33(45.21)	4(6.78)		
III	30(45.10)	55(93.22)		

### Comparison of MRI characteristics between KHE and KMP groups

In the KHE group, 63.01% of the lesions invaded the muscle layer, compared to 79.66% in the KMP group, significantly lower in the KHE group (*P* < 0.05). The proportion of lesions exhibiting flow voids in the KHE group was 35.62%, compared to 50.85% in the KMP group, with statistically significant differences between the two groups (*P* < 0.05) (Table 3).

**Table 3:** Comparison of MRI characteristics between the two groups of cases

Characteristic	KHE group (*n* = 73)	KMP group (*n* = 59)	χ^2^/t/Z	*P* value
Lesion boundary			3.704	0.125
Clear	2(2.74)	4(6.78)		
Blurry	71(97.26)	55(93.22)		
Invasion of the muscle layer	46(63.01)	47(79.66)	2.904	0.021*
Flow void effect	26(35.62)	30(50.85)	2.105	0.016*
Subcutaneous tissue edema	64(87.67)	51(86.44)	1.904	0.103
T1WI signal			4.902	0.070
Low signal	65(89.04)	48(81.36)		
Isointensity	7(9.59)	9(15.25)		
High signal	1(1.37)	2(3.39)		
T2WI signal			3.801	0.142
Low signal	2(2.74)	4(6.78)		
Isointensity	6(8.22)	39(5.08)		
High signal	65(89.04)	52(88.14)		

## Discussion

Kaposiform Hemangioen dothelioma (KHE) is an uncommon vascular tumor predominantly seen in infants. Cohort studies [13] have shown that 52.1% of KHE cases are identified at birth, and 91.8% manifest within the first year of life. In our study, 90.91% (120/132) of the children were diagnosed within their first year, aligning closely with previous findings. The International Society for the Study of Vascular (ISSVA) Anomalies categorizes KHE based on its growth characteristics as a tumor with “locally aggressive or borderline behavior.” In our study, 125 KHE cases presented as single lesions, with 7 cases exhibiting multiple lesions. Although there have been no reports of distant metastasis internationally, instances of multifocal KHE distribution [14] have been documented, mirroring the outcomes of this study.

Among pediatric vascular-origin diseases, KHE is relatively rare, and its nuances are often not well-understood by clinical pediatricians. However, KHE can lead to Kasabach-Merritt Phenomenon (KMP), resulting in severe clinical implications, underscoring the importance of early detection. The majority of affected children in this study showed cutaneous involvement, with extensive purple-red (56 cases) or red (55 cases) firm, warm nodules, distinctly different from common hemangiomas. These characteristics, combined with imaging features, facilitate a preliminary diagnosis. A minority of patients without skin involvement (21 cases) presented with firm, immobile subcutaneous nodules, which could be misidentified as other types of hemangiomas [15]. Given KHE’s rapid progression and local aggressiveness, especially in cases associated with KMP where bleeding is a possibility, biopsies should be approached with caution.

KMP is a clinical hallmark of KHE, characterized by decreased platelet count, reduced fibrinogen levels, elevated D-dimer levels, and a hypocoagulable state potentially leading to intermittent bleeding [16]. The incidence of KMP correlates positively with the tumor’s diameter. KHEs located in muscles, joints, or bones are less likely to trigger KMP due to limited growth space, which inhibits abnormal proliferation of vascular endothelial cells [17]. Other symptoms depend on the KHE’s location; bone involvement can lead to destruction and, in severe cases, pathological fractures, limiting limb movement and causing pain. If the pleura or pericardium is involved, pleural or pericardial effusion may occur; KHEs in the abdominal cavity or retroperitoneum can lead to secondary intestinal obstruction or hydronephrosis, and pancreatic KHEs may cause obstructive jaundice.

Ultrasound imaging of KHE reveals heterogeneous echoic masses within soft tissue, generally larger, irregular in shape, and poorly defined due to its invasive growth characteristics. The lesion’s center appears hypoechoic, with surrounding tissue often thickened and echo-enhanced due to tumor infiltration [18], presenting a distinctive feature. Few lesions may contain tubular anechoic areas internally. Color Doppler ultrasound demonstrates abundant blood flow within the lesion, classified as Adler grade III, displaying a characteristic branching pattern from deep to superficial layers. This study observed that the proportion of lesions extending into the fat layer and invading the muscle was lower in the KHE group compared to the KMP group, with smaller lesion diameters in the KHE group. The primary blood flow classification in the KHE group was grade II (45.21%), followed by grade III (45.10%), whereas in the KMP group, grade III was predominant (93.22%). Research by Liu et al. [19] also indicated that larger lesions, particularly those in KHE with KMP, suggest a higher likelihood of invading surrounding tissues or accompanying KMP, aligning with our findings and highlighting the need for close monitoring of platelet fluctuations in KHE patients.

KHE is invasive, with both focal and diffuse lesions on MRI appearing as irregular soft tissue masses with ragged edges, capable of invading surrounding fat, superficial fascia, deep fascia, muscle, and even bone [20]. All lesions in the children included in this study showed invasive characteristics, consistent with research conclusions. Furthermore, 63.01% of lesions in the KHE group invaded the muscle layer, compared to 79.66% in the KMP group, indicating a significantly lower rate in the KHE group. Contrast-enhanced MRI of KHE typically shows significant, persistent enhancement of the lesion, surrounded by abundant vasculature. In this study, the incidence of flow voids in the KHE group was lower than in the KMP group, likely related to the pathological features of the tumor’s vascular channels and thin-walled lymphatic vessels [21].

This study has limitations, including the similar age range of the cases, which does not provide direct evidence for the consistency of KHE imaging features across different age groups. The sample selection did not include cases in rare locations. Demonstrating consistent ultrasound and MRI characteristics of KHE across different age groups and rare locations could further enhance the clinical application value of ultrasound diagnosis for KHE.

## Conclusion

In conclusion, KHE is a clinically rare disease, with diagnosis primarily based on clinical presentation, histopathology, and immunohistochemical markers. Most cases involve KMP, leading to severe clinical outcomes, thus early diagnosis and treatment are crucial. KHE patients with KMP tend to have lesions that easily extend into the fat layer and invade the muscle layer, with larger lesion diameters, rich blood flow, and MRI images showing flow voids. Clinical diagnosis should include differentiation from other tumors.

## Data Availability

All data generated or analysed during this study are included in this article. Further enquiries can be directed to the corresponding author.

## References

[1] Ji Y, Chen S, Yang K, et al. Kaposiform hemangioendothelioma: current knowledge and future perspectives. Orphanet J Rare Dis. 2020;15(1):39. 10.1186/s13023-020-1320-1. 32014025 10.1186/s13023-020-1320-1 PMC6998257

[2] McDaniel CG, Adams DM, Steele KE, et al. Kaposiform lymphangiomatosis: diagnosis, pathogenesis, and treatment. Pediatr Blood Cancer. 2023;70(4):e30219. 10.1002/pbc.30219. 36683202 10.1002/pbc.30219 PMC10018800

[3] Johnson AB, Richter GT. Vascular anomalies. Clin Perinatol. 2018;45(4):737–49. 10.1016/j.clp.2018.07.010. 30396415 10.1016/j.clp.2018.07.010

[4] Brill R, Uller W, Huf V, et al. Additive value of transarterial embolization to systemic sirolimus treatment in kaposiform hemangioendothelioma. Int J Cancer. 2021;148(9):2345–51. 10.1002/ijc.33406. 33231291 10.1002/ijc.33406

[5] Qi Hongyan M, Lin Z, Jinzhe. Eight cases of Infantile Giant Hemangioma with Thrombocytopenia Syndrome. J Clin Pediatr Surg. 2008;7(06):47–8.

[6] Ji Y, Chen S, Xiang B, et al. Sirolimus for the treatment of progressive kaposiform hemangioendothelioma: a multicenter retrospective study. Int J Cancer. 2017;141(4):848–55. 10.1002/ijc.30775. 28486787 10.1002/ijc.30775

[7] Putra J, Gupta A. Kaposiform haemangioendothelioma: a review with emphasis on histological differential diagnosis. Pathology. 2017;49(4):356–62. 10.1016/j.pathol.2017.03.001. 28438388 10.1016/j.pathol.2017.03.001

[8] Chundriger Q, Tariq MU, Abdul-Ghafar J, et al. Kaposiform Hemangioendothelioma: clinicopathological characteristics of 8 cases of a rare vascular tumor and review of literature. Diagn Pathol. 2021;16(1):23. 10.1186/s13000-021-01080-9. 33722245 10.1186/s13000-021-01080-9 PMC7962213

[9] Ritai S. Imaging and pathological progress of Kaposiform Hemangioendothelioma. Chin J Aesthetic Med. 2020;29(08):186–9. 10.15909/j.cnki.cn61-1347/r.003906.

[10] Goyal P, Alomari AI, Kozakewich HP, et al. Imaging features of kaposiform lymphangiomatosis. Pediatr Radiol. 2016;46(9):1282–90. 10.1007/s00247-016-3611-1. 27053281 10.1007/s00247-016-3611-1

[11] Ding Y, Wang Z, Xu P, et al. MRI-based radiomics in distinguishing Kaposiform hemangioendothelioma (KHE) and fibro-adipose vascular anomaly (FAVA) in extremities: a preliminary retrospective study. J Pediatr Surg. 2022;57(7):1228–34. 10.1016/j.jpedsurg.2022.02.031. 35418319 10.1016/j.jpedsurg.2022.02.031

[12] Diagnosis and Treatment Guideline for Hemangiomas and Vascular Malformations. (2024 Version). Journal of Tissue Engineering and Reconstructive Surgery. 2024;20(01):1–50.

[13] Ji Y, Yang K, Peng S, et al. Kaposiform haemangioendothelioma: clinical features, complications and risk factors for Kasabach-Merritt phenomenon. Br J Dermatol. 2018;179(2):457–63. 10.1111/bjd.16601. 29603128 10.1111/bjd.16601 PMC11032113

[14] Nakaya T, Morita K, Kurata A, et al. Multifocal kaposiform hemangioendothelioma in multiple visceral organs: an autopsy of 9-day-old female baby. Hum Pathol. 2014;45(8):1773–7. 10.1016/j.humpath.2014.03.019. 24931465 10.1016/j.humpath.2014.03.019

[15] Drolet BA, Trenor CC 3rd, Brandão LR, et al. Consensus-derived practice standards plan for complicated Kaposiform hemangioendothelioma. J Pediatr. 2013;163(1):285–91. 10.1016/ j.jpeds.2013.03.080. 23796341 10.1016/j.jpeds.2013.03.080

[16] Goldenberg M, Shiel M, Subramanian S, et al. Splenic kaposiform hemangioendothelioma presenting as insidious consumptive coagulopathy. Am J Hematol. 2021;96(12):1708–14. 10.1002/ajh.26370. 34622468 10.1002/ajh.26370

[17] Peng S, Xia C, Yang K, et al. Kaposiform haemangioendothelioma: magnetic resonance imaging features in 64 cases. BMC Pediatr. 2021;21(1):107. 10.1186/s12887-021-02573-8. 33657997 10.1186/s12887-021-02573-8 PMC7927413

[18] Calvo-Garcia MA, Kline-Fath BM, Adams DM, et al. Imaging evaluation of fetal vascular anomalies. Pediatr Radiol. 2015;45(8):1218–29. 10.1007/s00247-014-3248-x. 25492302 10.1007/s00247-014-3248-x

[19] Liu Yifei Y, Jianjun S, Kaikai, et al. The Ultrasonographic Features and pathological manifestations of Kaposiform Hemangioendothelioma. J Med Imaging. 2023;33(05):862–5.

[20] Li T, Huanhuan Z, Xiujun Y. CT and MRI manifestations of soft tissue Kaposiform Hemangioendothelioma in Children. Chin J Med Imaging Technol. 2024;40(06):898–901. 10.13929/j.issn.1003-3289.2024.06.021.

[21] Ryu YJ, Choi YH, Cheon JE, et al. Imaging findings of Kaposiform Hemangioendothelioma in children. Eur J Radiol. 2017;86:198–205. 10.1016/j.ejrad.2016.11.015. 28027747 10.1016/j.ejrad.2016.11.015

